# Electrochemical insights into manganese–cobalt doped α-Fe_2_O_3_ nanomaterial for cholesterol detection: a comparative approach

**DOI:** 10.1039/d5ra04373e

**Published:** 2025-09-18

**Authors:** Sushmitha S, Subhasmita Ray, Lavanya Rao, Mahesha P. Nayak, Karel Carva, Badekai Ramachandra Bhat

**Affiliations:** a Department of Chemistry, Catalysis and Materials Chemistry Laboratory, National Institute of Technology Karnataka Surathkal D. K. Karnataka 575 025 India ram@nitk.edu.in; b Department of Condensed Matter Physics, Faculty of Mathematics and Physics, Charles University Ke Karlovu 3 Prague 12116 Czech Republic

## Abstract

Herein, a self-assembled hierarchical structure of hematite (α-Fe_2_O_3_) was synthesized *via* a one-pot hydrothermal method. Subsequently, the nanomaterial was doped to obtain M_*x*_Fe_2−*x*_O_3_ (M = Mn–Co; *x* = 0.01, 0.05, and 0.1) at precise concentrations. An electrode was fabricated by coating the resulting nanocomposite onto a nickel foam (NF) substrate. Electrochemical characterization demonstrated the excellent performance of cobalt-doped α-Fe_2_O_3_, among which Co_0.05_Fe_0.95_O_3_ (CF5) exhibited a superior performance, showing a two-fold increase in sensitivity of 1364.2 μA mM^−1^ cm^−2^ (±0.03, *n* = 3) in 0.5 M KOH, a limit of detection (LOD) of ∼0.17 mM, and a limit of quantification (LOQ) of ∼0.58 mM. The Density Functional Theory (DFT) was performed to understand the doping prompting in the reduced bandgap. The fabricated electrode displayed a rapid response time of 2 s and demonstrated 95% stability, excellent reproducibility, and selectivity, as confirmed by tests with several interfering species. A comprehensive evaluation of the electrode's performance using human blood serum highlighted its robustness and reliability for cholesterol detection in clinical settings, making it a promising tool for clinical and pharmaceutical applications.

## Abstract 2

Cobalt-doped α-Fe_2_O_3_ nanostructures show enhanced sensitivity of 1364.2 μA mM^−1^ cm^−2^ (±0.03, *n* = 3), selectivity, and stability for nonenzymatic cholesterol detection, with strong clinical potential supported by DFT analysis and human serum sample.

## Graphical abstract

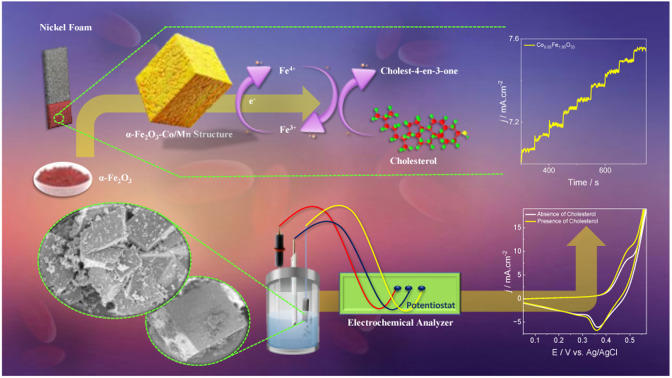

## 1. Introduction

Cardiovascular diseases (CVDs) are the leading cause of mortality worldwide, with growing public awareness since the 1980s regarding the risks associated with elevated blood cholesterol levels.^1^ Cholesterol, a unique waxy lipid, is essential for various bodily functions, necessitating its close monitoring.^2^ Numerous studies have proven that elevated cholesterol levels noticeably increase the risk of CVDs.^3^ In healthy individuals, the cholesterol levels are below 200 mg dL^−1^ (5.17 mM), and the levels around 240 mg dL^−1^ (6.21 mM) are associated with peripheral vascular diseases, insulin-dependent diabetes, hypertension, and cardiovascular conditions.^3–6^

Regular monitoring of cholesterol levels has become essential in contemporary healthcare, prompting the development of various methods to assess cholesterol concentrations accurately. Consequently, numerous techniques have emerged for the detection of cholesterol in biological and food samples. These include spectrophotometric methods,^7^ electrochemical approaches,^4^ high-performance liquid chromatography (HPLC),^8^ and enzymatic colorimetric techniques.^9^ Although these techniques demonstrate significant effectiveness in cholesterol sensing, they still possess certain limitations such as time-consuming processes, high cost, requirement of expensive equipment, and potential background noise.^10^ To tackle the problems based on these techniques, non-enzymatic biosensors that display strong thermal and chemical stability and good sensitivity and selectivity have gained a greater interest among researchers.^11^ To bridge the gap, integrating non-enzymatic biosensors and nanotechnology has resulted in the creation of nanocomposites that considerably enhance the electrochemical performance of cholesterol sensors.^12,13^ These advancements offer several predominant benefits, including improved selectivity, reduced cost, ease of handling, and rapid response times, rendering them highly effective for point-of-care testing.^14^

The functionality of biosensors hinges on the selection and development of nanostructured materials.^15^ To develop electrochemically active substances for non-enzymatic sensing applications, various nanomaterials, including zero-dimensional (0D) nanomaterials,^16^ one-dimensional (1D) nanowires^17^ and tubes,^18^ two-dimensional (2D) metals/metal oxides,^19,20^ graphenes,^21^ and three-dimensional (3D) nanoflowers,^22,23^ cubes,^24^ rods^25,26^ and spheres,^27^ have been analyzed.

Although advanced materials remain the primary focus of research, there is an increasing interest in simpler nanocomposites that demonstrate comparable sensitivity and electrochemical performance. In recent years, transition metal oxides including ZnO, CuO, SnO_2_, Fe_2_O_3_, Ag_2_O, WO_3_, NiO_2_, and V_2_O_5_ have been extensively investigated for their potential in non-enzymatic sensing applications.^6,28–30^ These materials exhibit high efficacy owing to their metal centers and greater surface areas, thereby enhancing the density of electrochemically active sites in alkaline environments.^16,31–36^ In the past few years, doping metal oxides has been shown to boost electrochemical performance and photocatalytic activity and improve electrical and photoelectrochemical features by boosting the charge carrier density and conductivity.^37^

Currently, activated carbon derived from *Piper nigrum* is being synthesized and combined with α-Fe_2_O_3_, followed by modification with a carbon paste electrode (APC-Fe_2_O_3_/CPE)^38^ exhibiting high sensitivity with a linear range of 25 nM to 300 nM, an LOD of 8 nM, and an LOQ of 26 nM. Additionally, electrolyte-gated transistor-based biosensors utilizing α-Fe_2_O_3_ decorated with ZnO nanorods demonstrated a broad linear range of 0.1 to 60 mM and a sensitivity of 37.34 μA mM^−1^ cm^−2^.^39^ Moreover, the phase transition of Fe_3_O_4_ to α-Fe_2_O_3_ was achieved *via* electrophoretic film deposition on ITO-coated glass plates, which exhibited a sensitivity of 193 nA mg^−1^ dl cm^−2^ with a linear range and response time of 25–500 mg dl^−1^ and 60 s, respectively.^40^ Another study on the electrochemical behavior of citrate-modified β-cyclodextrin (CIT-BCD) and Fe_3_O_4_ synthesized *via* the coprecipitation method (CIT-BCD@Fe_3_O_4_) reported a linear range of 0 to 100 μM and an LOD of 3.93 μM.^41^

A bimetallic nanocomposite glassy carbon electrode combined with a Cu_2_O/MoS_2_ nanohybrid, which demonstrated a sensitivity of 111.74 μA μM^−1^ cm^−2^ with an LOD of 2.18 μM and a linear range spanning from 0.1 to 180 μM, was showcased as an effective alternative for cholesterol sensing.^42^ Additionally, utilizing low-cost galvanic deposition, a ZnO/WO_3_ composite demonstrated a sensitivity of 176.6 μA cm^−2^ mM^−1^ with a linear range of 0–320 μM and an LOD of 5.5 nM.^43^ Another study on the electrochemical study of NiO/CuO nanocomposites synthesized by an electrospinning method reported a sensitivity of 10.27 μA mM^−1^ cm^−2^, a linear range of 0.8 to 6.5 mM and an LOD of 5.9 μM.^44^ Despite these advancements, challenges such as cost-effectiveness, lifespan, stability, and sensitivity to pH and temperature variations complicate the use of complex molecular sensors. To address these issues, our study aims to develop a transition metal-doped metal oxide cholesterol sensor to enhance selectivity, sensitivity, and stability while reducing costs. This study clearly shows the strategic comparative synthesis of bimetallic M_*x*_Fe_2−*x*_O_3_ (M = Mn–Co), and to the best of our knowledge, this is the first comparative study investigating a nonenzymatic cholesterol sensor.

The primary goal of this work was to synthesize α-Fe_2_O_3_*via* a hydrothermal approach and subsequently implement doping M_*x*_Fe_2−*x*_O_3_ (M = Mn–Co, and *x* = 0.01, 0.05, and 0.1). The doping concentrations of 1%, 5%, and 10% were selected to systematically investigate the variations in electronic structure, redox activity, and conductivity induced by doping. The obtained materials were fabricated on NF, and the electrodes were evaluated for sensitivity, LOD, LOQ, response time, and linear range. The comparison of the doped materials was intended to choose the most promising dopants, which were then subjected to a comparison analysis of their properties for biosensing applications. Electrochemical characteristics were assessed by Cyclic Voltammetry (CV), chronoamperometry (CA), Differential Pulse Voltammetry (DPV), Electrochemical Impedance Spectroscopy (EIS), and the evaluation of electrochemical active surface area (ECSA). The selected material was further analyzed for its application in blood serum cholesterol detection.

## 2. Experimental details

### 2.1 Chemicals and materials

Nickel foam (NF; thickness 0.5 mm, 99.9% purity) was purchased from Global Nanotech Mumbai. Iron(iii) chloride tetrahydrate (FeCl_3_·4H_2_O, 99% Purity), dopamine hydrochloride (DA), poly(vinylidene fluoride) (PVDF), Triton™ X-100, and urea (CO(NH_2_)_2_, 98% purity) were procured from Sigma-Aldrich, Germany. Manganese(ii) acetate tetrahydrate ((CH_3_COO)_2_Mn·4H_2_O, 98.5% Extra Pure), cobalt(ii) acetate tetrahydrate ((CH_3_COO)_2_Co·4H_2_O, 98.5% Extra Pure), potassium hydroxide pellets (KOH, 85% Extra Pure), uric acid ((UA), C_5_H_4_N_4_O_3_, 99% AR), cholesterol (C_27_H_46_O, 97% extra pure), l-ascorbic Acid ((AA), C_6_H_8_O_6_, 99% extra pure), *N*-methyl-2-pyrrolidone (NMP) (C_5_H_9_NO, 98% Purity), potassium chloride (KCl, 99% Purity), and sodium chloride (NaCl, 99.5% Purity) were purchased from LOBA Chemie Pvt. Ltd. Ultra-pure Milli-Q water (Elga Veolia) was utilized throughout the experiment. All chemicals were of analytical grade and used without filtering.

### 2.2 Synthesis of M_*x*_Fe_2−*x*_O_3_ (M = Mn–Co and *x* = 0.01, 0.05, and 0.1)

**Fig. 1:**
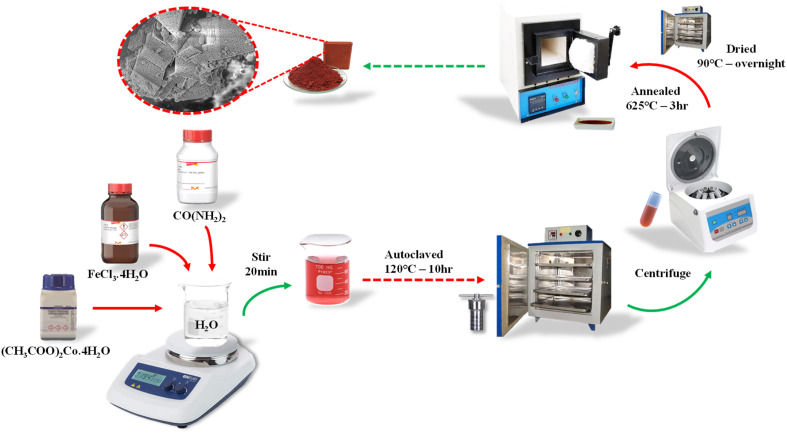
Schematic representation of the synthesis of M_*x*_Fe_2−*x*_O_3_ (M = Mn–Co and *x* = 0.01, 0.05, and 0.1).

α-Fe_2_O_3_ and M_*x*_Fe_2−*x*_O_3_ nanostructures were synthesized by preparing a homogeneous solution of 0.1 M FeCl_3_·4H_2_O and 0.1 M CO(NH_2_)_2_ dissolved in 80 mL of Milli-Q water for 30 minutes. To this solution, calculated quantities of Mn and Co precursors (with *x* = 0.01, 0.05, and 0.1) were added. The resulting mixture was loaded into a 100 mL autoclave and subjected to hydrothermal processing at 120 °C for 10 hours. Once the reaction was completed, the mixture was allowed to reach room temperature and collected by centrifugation. The precipitate was washed twice with Milli-Q water, followed by a single wash with ethanol. Once washed, the products were dried in an oven at 90 °C overnight and then annealed at 625 °C for 3 hours. A schematic representation of the synthesis process of M_*x*_Fe_2−*x*_O_3_ is presented in Fig. 1.

The resulting products were designated with specific names based on their composition as Co_0.01_Fe_1.99_O_3_ (CF1), Co_0.05_Fe_1.95_O_3_ (CF5), Co_0.1_Fe_1.90_O_3_ (CF10), Mn_0.01_Fe_1.99_O_3_ (MF1), Mn_0.05_Fe_1.95_O_3_ (MF5), and Mn_0.1_Fe_1.90_O_3_ (MF10).

### 2.3 Fabrication of the electrode

The synthesized nanocomposite comprising α-Fe_2_O_3_ and M_*x*_Fe_2−*x*_O_3_ (M = Mn–Co, and *x* = 0.01, 0.05, and 0.1) was fabricated on the NF electrode. Initially, accurately measured product samples were blended with PVDF in a proportion of 9 : 1 in a mortar and pestle. Subsequently, NMP was gradually added drop by drop till the mixture achieved paste consistency. The resulting paste was uniformly coated on a 1 × 1 cm^2^ area of priorly treated NF and dried in a vacuum oven for an extended period of 24 hours at 60 °C, and the quantity of product loaded was 10 ± 0.2 mg.

### 2.4 Material and electrochemical characterisations

The structural properties that enabled the identification and characterization of distinct phases and crystalline compositions of the material under research were analyzed using a Rigaku Miniflex 600 powder X-ray diffraction (XRD) device. The measurement covers a range of 5° to 90° and a scanning rate of 3° per minute with monochromatic Cu-Kα radiation of wavelength 0.154 nm. The crystallite size (*D*) was calculated based on the Debye–Scherrer equation (eqn (1)) ^45^:1
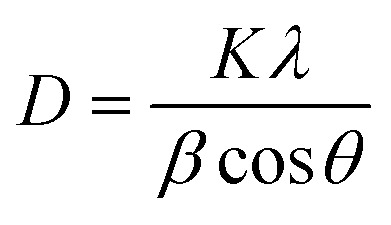
where *K* is the Scherrer constant, *λ* is the Cu-Kα radiation wavelength, *β* is the full width at half maximum (FWHM) of the peak, and *θ* is the Bragg angle. The phonon vibration modes were examined using a confocal Raman microscope integrated with a compact Raman spectrometer (Renishaw, UK) with an objective lens magnification of ×50. The optical absorption spectra were recorded using an ultraviolet-near infrared spectrophotometer (UV-vis-NIR, Lambda 950, PerkinElmer, Singapore). The chemical composition of the synthesized sample was determined using a Thermo Fisher Scientific ESCALAB Xi + X-ray Photoelectron Spectrophotometer (XPS) with an Al Kα X-ray source (1486.7 eV) for the analysis. Morphological imaging was performed using a field emission scanning electron microscope (FESEM) (7610FPLUS, Jeol, Japan) equipped with an energy-dispersive X-ray spectrometer (EDAX), enabling a thorough investigation of the morphological characteristics.

The electrochemical characterization was conducted using an Autolab PGSTAT204 electrochemical workstation. CV experiments employed a three-electrode configuration, with the synthesized material coated on a NF serving as the working electrode (WE), covering an area of 1 × 1 cm^2^. A saturated Ag/AgCl electrode (with potassium chloride) served as the reference electrode (RE), while a platinum electrode functioned as the counter electrode (CE), both operating within a 0.5 M KOH electrolyte solution. CV analysis was performed within a potential window of 0 to 0.75 V, at scan rates ranging from 5 to 120 mV s^−1^. CA studies involved sequentially adding a cholesterol solution into the electrolyte under continuous stirring, maintained at a constant applied potential of +0.55 V *vs.* Ag/AgCl. DPV was executed over a potential range of 0.35 to 0.65 V *vs.* Ag/AgCl at a scan rate of 10 mV s^−1^ and a pulse amplitude of 50 mV. The ECSA was evaluated by varying the scan rates from 5 to 75 mV s^−1^ within a potential range. The double-layer capacitance (*C*_dl_) was determined by plotting Δ*j* (where Δ*j* = *j*_*a*_ −*j*_*c*_) against the scan rate. The accurate surface area was then calculated using the formula *C*_dl_/*C*_s_,^46^ where *C*_s_ is the capacitance of an atomically smooth surface, taken as 40 μF cm^−2^.^47,48^ The sensitivity was evaluated from the calibration curve obtained by plotting current *versus* concentration, as expressed by the corresponding equation (eqn (2)):2
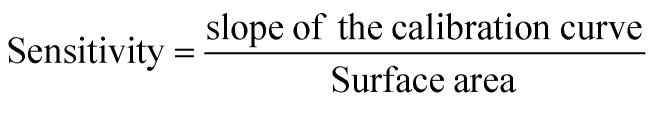


The formulas used to calculate the LOD and LOQ are as follows (eqn (3) and (4)):3
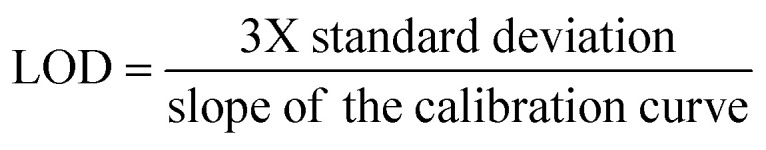
4
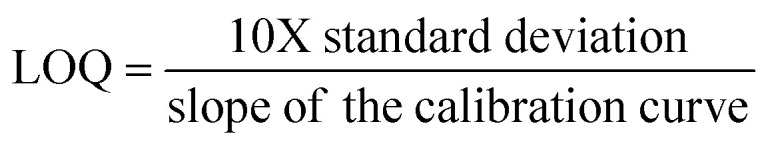
In order to ensure reproducibility, each test was carried out three times (*n* = 3), and the mean ± standard deviation was employed to depict the results. Statistical significance was regarded as a variation with *p* < 0.03 at the level of confidence set at 97%.

## 3. Results and discussion

The phase purity and composition of α-Fe_2_O_3_ and M_*x*_Fe_2−*x*_O_3_ (M = Mn–Co, and *x* = 0.01, 0.05, and 0.1) were analyzed using a powder XRD. Fig. S1(a–c) present the XRD patterns of α-Fe_2_O_3_, and M_*x*_Fe_2−*x*_O_3_, revealing a clear match with the JCPDS standard (No. 33-0664) and confirming its rhombohedral hexagonal phase. The non-appearance of additional impurity diffraction peaks in the XRD spectra confirms the pristine nature of α-Fe_2_O_3_. The diffraction peaks at 2*θ* angles of 23.83°, 32.99°, 35.46°, 40.69°, 49.47°, 54.08°, 57.14°, 62.57°, and 63.87° correspond to the (012), (104), (110), (113), (024), (116), (018), (214), and (300) planes of α-Fe_2_O_3_,.^49^

**Fig. 2:**
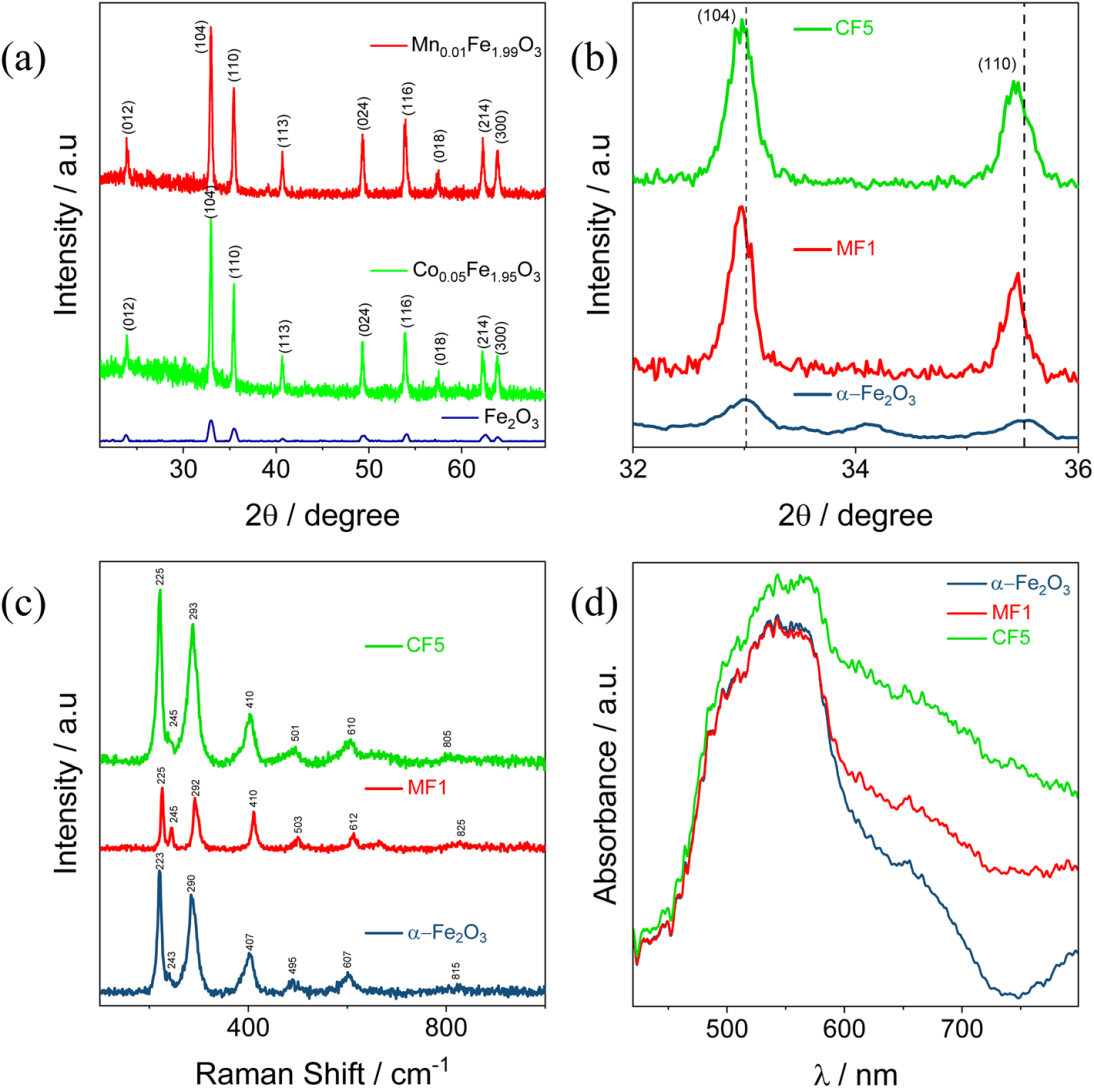
(a) XRD spectra of α-Fe_2_O_3,_ CF5, and MF1; (b) shift of the (104) and (110) XRD peaks to lower diffraction angles due to the incorporation of substituents; (c) Raman spectra analysis of α-Fe_2_O_3,_ MF1, and CF5, respectively; and (d) UV-Vis absorption profiles of α-Fe_2_O_3,_ CF5, and MF1, respectively.

Fig. 2(a and b) present the XRD spectra of α-Fe_2_O_3_, CF5, and MF1 materials under investigation. A notable shift in the diffraction peaks toward lower angles, along with changes in peak intensity, confirms successful doping in the respective host structures. This shift is ascribed to the lattice distortion induced by the incorporation of Co^3+^ (0.61 Ȧ) and Mn^3+^ (0.645 Ȧ), which possess smaller ionic radii compared to Fe^3+^ (0.645 Ȧ), into the α-Fe_2_O_3_ lattice. The crystallite size, estimated using the Debye–Scherrer equation (eqn (1)) concerning the (104) diffraction plane, revealed an initial size of 16.18 nm for pure α-Fe_2_O_3_. In contrast, the doped samples MF1 and CF5 exhibited significantly larger crystallite sizes of 32.69 nm and 37.39 nm, correspondingly. This increase in crystallinity introduces defects in the α-Fe_2_O_3_ structure, enhancing the electron transfer kinetics, which accounts for the superior electrochemical performance of CF5/NF.^50^

The Raman spectra analysis depicted in Fig. 2(c) provides valuable insights into the structural characteristics of nanoparticles for α-Fe_2_O_3_, MF1, and CF5. Additional Raman spectra data for the remaining dopants are presented in Fig. S4(a and b). The vibrational spectra of pure α-Fe_2_O_3_ exhibit two distinct peaks at 223 and 495 cm^−1^ corresponding to A_1g_ modes and five peaks at 243, 290, 407, 607, and 815 cm^−1^ corresponding to *E*_g_ modes. These seven peaks collectively indicate the characteristic vibrational modes of the α-Fe_2_O_3_ structure.^49^ Notably, the absence of extra peaks signifies purity and confirms that α-Fe_2_O_3_ nanoparticles are produced without contaminants. The Raman spectra of MF1 and CF5 closely resemble those of pure α-Fe_2_O_3_, underscoring that the doped nanoparticles maintain their unique hematite structure. Despite the incorporation of Mn and Co dopants, the structural characteristics of α-Fe_2_O_3_ remain unchanged, as evidenced by the consistent presence of these features across the doped samples.

The UV-vis spectra of α-Fe_2_O_3_, CF5, and MF1 were measured in terms of absorbance, as illustrated in Fig. 2(d). The band edge absorption for α-Fe_2_O_3_ typically occurs in the 520–565 nm wavelength range. In this study, the compound exhibited strong absorption at 542 nm, with CF5 and MF1 also demonstrating prominent peaks within this range. The band gap energies of the materials were evaluated through the Kubelka–Munk function *f*(R)^2^ plotted against energy (eV) by utilizing reflectance spectra depicted in Fig. S4(c). Linear extrapolation at *f*(R)^2^ = 0 estimated the resulting band gap values: α-Fe_2_O_3_ (2.05 eV) > MF1 (2.02 eV) > CF5 (1.9 eV) (Fig. S4(d)). The observed reduction in the bandgap can be attributed to the formation of additional energy levels proximate to the valence band edge, which reduces the energy requirement for electronic transitions from valence to conduction bands.^51,52^

**Fig. 3:**
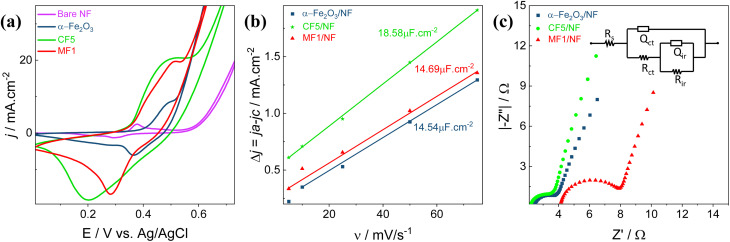
(a) Comparative analysis of CVs of bare NF, α-Fe_2_O_3_/NF, CF5/NF, and MF1/NF in 0.5 M KOH at a scan rate of 50 mV s^−1^; (b) ECSA plot of α-Fe_2_O_3_/NF, CF5/NF, and MF1/NF; and (c) Nyquist plots of α-Fe_2_O_3_/NF, CF5/NF, and MF1/NF, respectively.

The electrocatalytic performance of bare NF, α-Fe_2_O_3_/NF, and M_*x*_Fe_2−*x*_O_3_/NF (M = Mn–Co, and *x* = 0.01, 0.05, and 0.1) was evaluated by maintaining a scan rate of 50 mV s^−1^ over a potential window of 0 V to +0.75 V *vs.* Ag/AgCl using CV measurements in 0.5 M KOH, as depicted in Fig. S2(a–c). Among the doped materials, CF5/NF and MF1/NF demonstrated superior performance, which is demonstrated in Fig. 3(a). Notably, among the three electrodes, CF5/NF proved superior electrocatalytic activity, which is due to cobalt doping endowing more active sites to the α-Fe_2_O_3_ surface, making it easier for reactant molecules to adsorb and activate. Furthermore, cobalt ions can change the electronic structure of α-Fe_2_O_3_, resulting in improved charge transfer kinetics and catalytic activity.^53^ This is evident from the anodic and cathodic peaks observed during redox reactions. To elucidate the surface-active sites linked to the number of electrons exchanged primarily in the oxidation process, a detailed study of the CV profiles of α-Fe_2_O_3_/NF, CF5/NF, and MF1/NF, are illustrated in Fig. S3(a–c). The number of electron transfers for the three primary materials was determined to be 0.6 × 10^19^ for α-Fe_2_O_3_/NF, 4.53 × 10^19^ for MF1/NF, and 12.19 × 10^19^ for CF5/NF.^54,55^ The present study highlights that within the three materials, CF5/NF exhibits the highest electron transfer rate during the oxidation process, illustrating the better electrocatalytic performance of CF5/NF in alkaline electrolytes.

Unlike the BET method, the ECSA analysis entails the complete immersion of the material electrode in the electrolyte, enabling a precise assessment of its surface area activity, and the ECSA results of all the synthesised materials are depicted in Fig. S5(a–g).^48,54^Fig. 3(b) reveals that α-Fe_2_O_3_/NF, MF1/NF, and CF5/NF possessed 2*C*_dl_ values of 14.54 μF cm^−2^, 14.69 μF cm^−2^, and 18.58 μF cm^−2^, accordingly. The ECSA results correlate with the genuine surface areas of each electrode as 0.182 cm^2^, 0.184 cm^2^, and 0.232 cm^2^ in a respective manner. Furthermore, a detailed evaluation of the surface area calibration plot and calculation are enclosed in Fig. S5 (h, i) and Table S1. The electrocatalytic performance results demonstrate that the CF5/NF electrode possesses the highest ECSA activity, indicating its superior potential for electrocatalytic applications due to an increased number of active sites.

To assess the efficiency of electrical charge transfer, EIS was performed, and the results were interpreted through Nyquist plots, as presented in Fig. 3(c), to evaluate the resistance and capacitance of the material. For a more detailed interpretation, the Nyquist plots were modeled with the equivalent circuit *R*_s_(*Q*_ct_(*R*_ct_(*Q*_ce_*R*_ce_))), with the fitted parameters provided in Table S2. The electrolyte resistance between the working and counter electrodes, represented by solution resistance (*R*_s_), was determined from the *x*-intercept of the semicircle at high frequencies. Additionally, the electrode–electrolyte interface impedance, comprising parallel elements *Q*_ct_ and *R*_ct_, was included in the model. The internal resistance of the catalyst was represented by parallel elements *R*_ir_ and *Q*_ir_ in series with *R*_ct_. Notably, the semicircle for CF5/NF displayed a smaller radius, indicating a reduction in internal and charge transfer impedance. This is also confirmed by the Mott–Schottky plot in Fig. S4(e). This decrease in impedance and solution resistance correlates with the improved electrocatalytic performance observed for CF5/NF.^56^

**Fig. 4:**
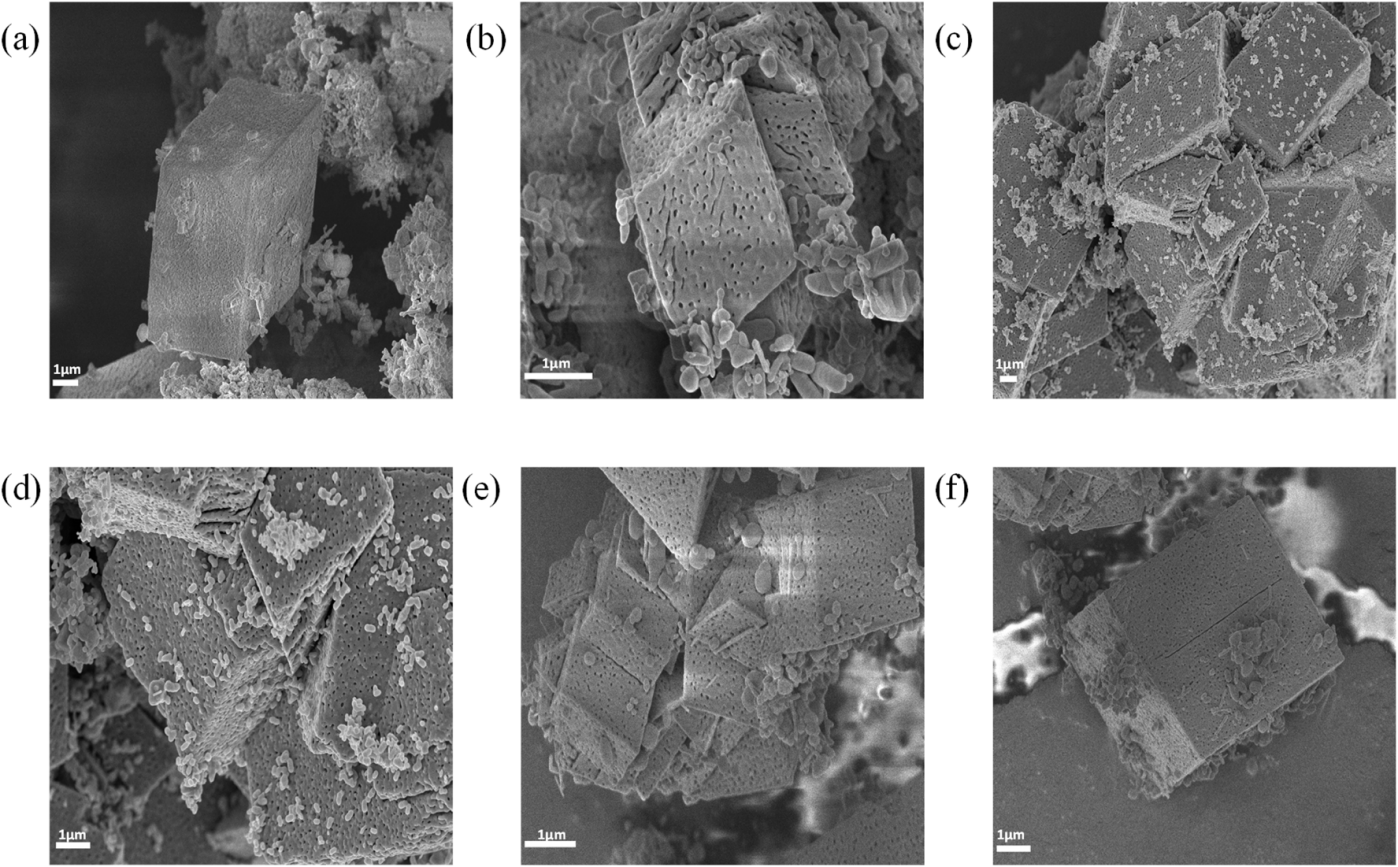
FESEM images of (a and b) α-Fe_2_O_3_; (c and d) CF5; and (e and f) MF1.

The nanocomposites were morphologically characterized by FESEM and EDAX. The FESEM images of α-Fe_2_O_3_, CF5, and MF1 nanoparticles are depicted in Fig. 4(a–f), respectively, illustrating their rhombus-like structure. The surface morphology is rough and porous, indicating the occurrence of α-Fe_2_O_3_ formation (Fig. 4(a, and b)). In CF5 and MF1, the incorporation of dopants into the lattice structure of α-Fe_2_O_3_ results in the formation of small granule-like structures on the surface of the framework; however, no surface defects are present within the lattice. Significantly, this observation implies that the α-Fe_2_O_3_ structure is not altered in CF5 and MF1.

In Fig. S6(c–e), the EDAX spectra reveal the images showcasing a relatively uniform distribution of Fe and O components throughout the sample, confirming the homogeneity of the synthesized materials.^57^ The low-intensity profiles observed for the Co and Mn dopants, in conjunction with the base elements, validate the effective incorporation of Co and Mn into the α-Fe_2_O_3_ matrix. Furthermore, EDAX analysis shows the existence of carbon (C) peaks at around 0.2 keV, which is attributed to the carbon tape used for sample handling during SEM and EDAX measurements.

**Fig. 5:**
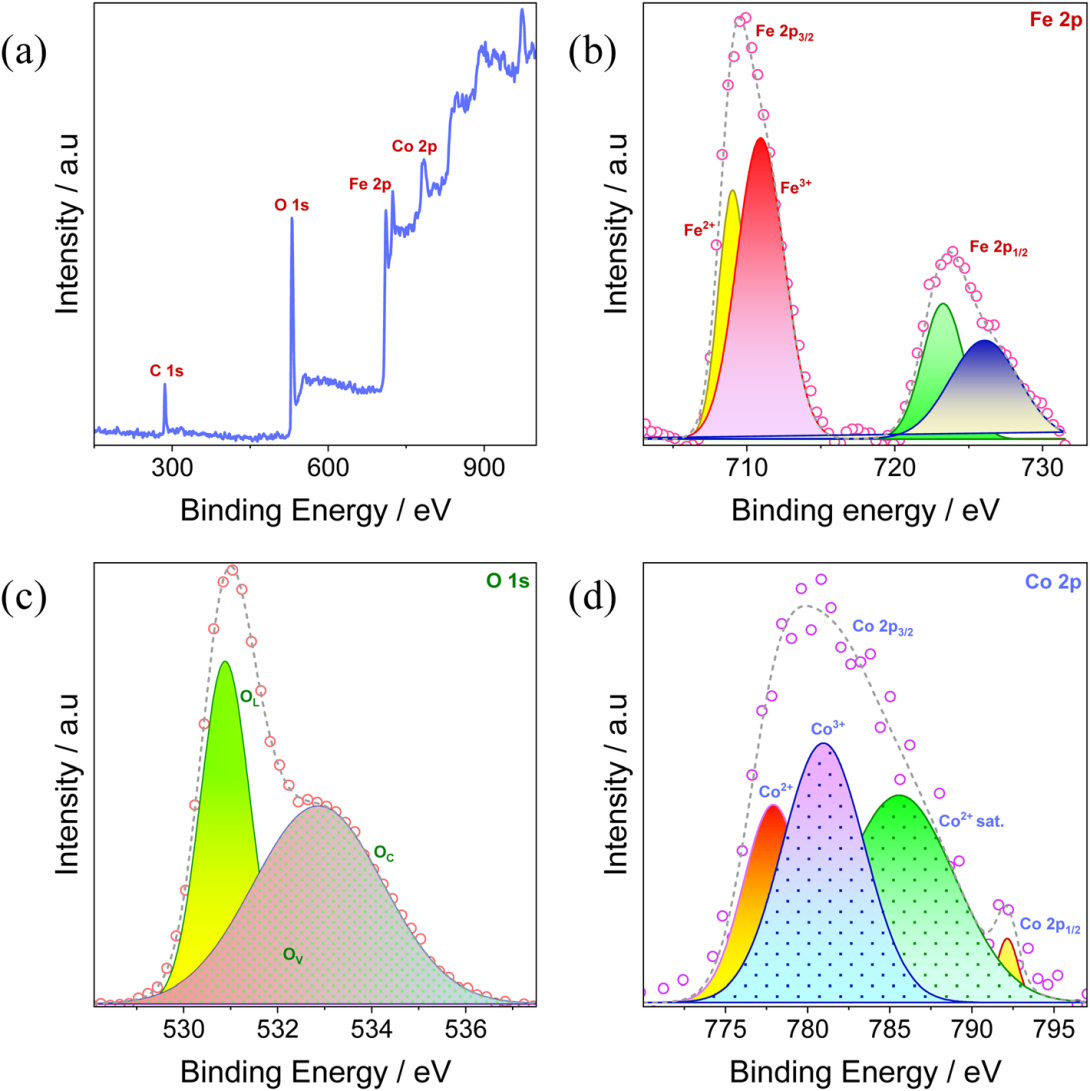
XPS spectrum of CF5: (a) survey; (b) Fe 2p; (c) O 1s; and (d) Co 2p.

XPS spectra was used to validate the surface chemical environment and oxidation state of CF5. Fe, O, Co, and C elements were identified on the surface of CF5, based on the survey scan (Fig. 5a) carried out over an energy range of 100–1000 eV, with no observable traces of contaminants. Two distinguished peaks, individually splitting into doublets, were observed in the high-resolution Fe 2p XPS spectra (Fig. 5b), demonstrating spin–orbit coupling in α-Fe_2_O_3_. In particular, the peaks at 723.27 eV and 726.06 eV were found to correspond to Fe^2+^ 2p_1/2_ and Fe^3+^ 2p_1/2_, whereas the peaks at 709 eV and 710.92 eV correlated with the binding energies of Fe^2+^ 2p_3/2_ and Fe^3+^ 2p_3/2_, respectively. The combined presence of Fe^2+^ and Fe^3+^ oxidation states points to a complex surface chemistry that may be impacted by dopant ion electron donation and oxygen vacancies from high-temperature annealing, among other factors.^58^

The clear peaks for the O 1s spectra of CF5 in Fig. 5c indicate the appearance of three distinct forms of surface oxygen species, which are represented by peaks at 530.87 eV involved with iron–oxygen bonding represented by the lowest energy peak, which is associated with lattice oxygen (O_L_), 531.63 eV at the intermediate energy level, oxygen vacancies (O_v_) appear as chemisorbed oxygen species, and 532.87 eV the final peak with the highest energy related to adsorbed oxygen species (O_c_). The obtained results were consistent with the findings previously reported in the literature.^59,60^

Similarly, the Co 2p XPS spectrum in Fig. 5d shows an obvious peak at 780 eV, which implies the presence of Co in the CF5 lattice. This peak exhibits additional subdivision, with Co 2p_3/2_ demonstrating three distinct peaks that show changes in the cobalt oxidation state. In particular, the Co^2+^ peak appears at around 777.88 eV, while the Co^3+^ peak is detected at 780.94 eV. Furthermore, a satellite peak of Co^2+^ is seen at 785.61 eV, which could be ascribed to plasmons, energy loss processes, or shake-ups. In addition, the cobalt 2p_1/2_ peak is observed at 792.16 eV, which adds to our understanding of the entire span of cobalt's oxidation states in the CF5 lattice.^61,62^

In this study, the Vienna *Ab initio* Simulation Package (VASP) was used to perform first-principles DFT simulations in order to examine the electronic structure and geometrical optimizations of both pristine and CF5 structures.^63–65^ Exchange-correlation interactions were described using the Perdew–Burke–Ernzerhof (PBE) functional in the generalized gradient approximation (GGA), while electron–ion interactions were correctly represented using the projector augmented wave (PAW) approach.^66,67^ To maintain a compromise between accuracy and computing economy, a plane-wave basis set with a kinetic energy cutoff of 520 eV was taken into consideration. For self-consistent computations, a *Γ*-centered 3 × 3 × 1 *k*-point mesh was used to sample the Brillouin zone. Using a U value of 5.0 eV based on the Dudarev and Botton technique,^68^ the Hubbard U correction was implemented within the GGA + U framework to account for strong electron correlations due to the presence of localized d-electrons in Fe and Co atoms. To minimize the force on each atom to less than 0.01 eV Ȧ^−1^, structural optimizations were performed. The energy convergence criterion between subsequent self-consistent stages was set at 1 × 10^−5^ eV. After building the bulk haematite structure to analyze its structural and electrical characteristics, two Fe atoms were swapped out for Co atoms in a supercell structure consisting of 80 atoms to study the effects of Co doping. A thorough examination of the structural and electrical properties of both pristine and CF5 structures was made possible by these computational methods.

### 3.1. Structural properties

**Fig. 6:**
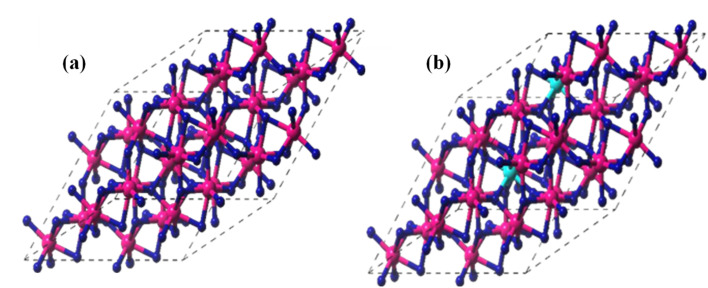
Optimized geometry of (a) Fe_2_O_3_ and (b) CF5.

DFT computations were used to examine the structural characteristics of pristine α-Fe_2_O_3_ and CF5, as shown in Fig. 6(a and b). Fe atoms occupy octahedral positions inside a hexagonal close-packed lattice of oxygen (O) atoms in α-hematite's rhombohedral structure (R-3c). For pristine α-Fe_2_O_3_, the optimized lattice parameters were found to be *a* = 5.44 Ȧ and *α* = 55.270°, which are values that closely match previous theoretical research and experimental results. The haematite structure's intrinsic cation–anion interactions are reflected in the Fe–O bond lengths within the octahedral units, which vary from 1.96 Ȧ to 2.10 Ȧ.

Minimal structural changes are brought about by the addition of 5% cobalt substitution, in which Co^3+^ ions take the place of Fe^3+^ ions. Optimal values of *a* = 5.42 Ȧ and *α* = 55.270° were obtained by a modest contraction of the lattice parameters because of the lesser ionic radius of Co^3+^ (roughly 0.61 Ȧ) compared to Fe^3+^ (about 0.645 Ȧ). The unit cell volume decreases as a result of this little drop in lattice characteristics, indicating that cobalt was successfully incorporated into the haematite lattice without causing significant structural deformities.

Additionally, there are minor variations in the bond lengths between the Co-substituted Fe–O octahedra and pure Fe–O bonds. The Co–O distances, which range from 1.95 Ȧ to 1.97 Ȧ, are marginally shorter than the comparable Fe–O bonds. The stronger Co–O interactions brought about by the more localized electronic character of the Co 3d states are responsible for this change.

### 3.2 Electronic properties of α-Fe_2_O_3_ and CF5

The electronic structure of a sensing material plays a key role in defining its conductivity, charge transfer efficiency, and surface reactivity, all of which directly influence its sensing ability. In biosensing applications, α-Fe_2_O_3_ is a promising material because of its surface activity, tunable bandgap,^69–71^ and a stable semiconducting nature. However, its efficiency is limited by its weak electrical conductivity and comparatively high bandgap. In order to alter the electrical structure and possibly lower the bandgap, introduce mid-gap states, and improve charge carrier mobility, Co doping was investigated.

Co-incorporation's effects on the material's electronic conductivity and surface interaction potential can be understood by examining the density of states (DOS) and band structure of both pure and CF5. Since enhanced conductivity and charge transfer capacities can improve the sensor's responsiveness, this information is crucial for optimizing hematite-based materials for cholesterol detection. The creation of more effective biosensors is guided by the fundamental insights into the material's potential as an active sensing element that the electronic structure analysis offers.

### 3.3 Band structure analysis

**Fig. 7:**
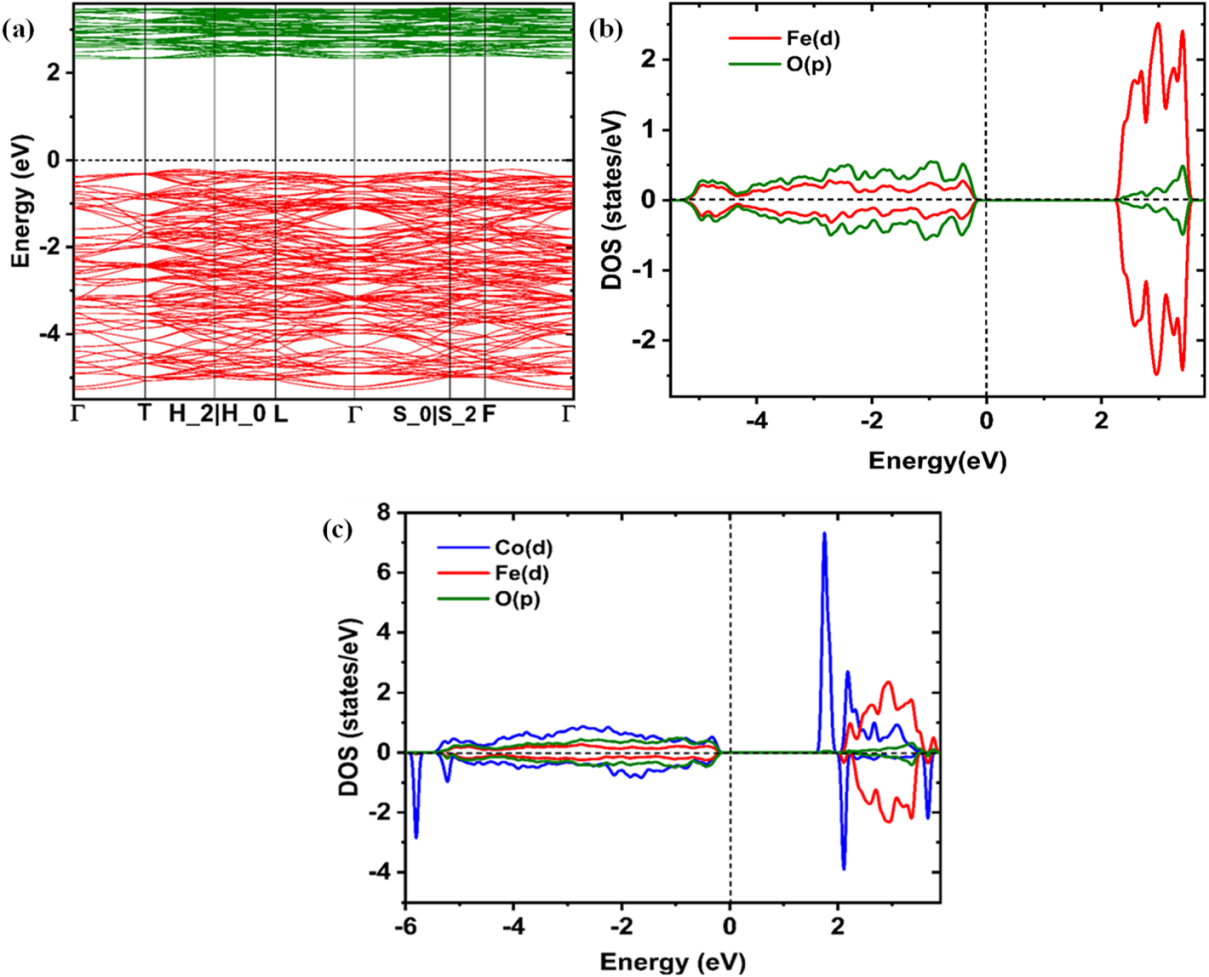
(a) Electronic band structure of α-Fe_2_O_3_ in the Brillouin zone of the rhombohedral cell, emphasizing the band gap along the high-symmetry path *Γ*-T-H_2_|H_0_-L-*Γ*-S_0_|S_2_-F-*Γ*. (b) α-Fe_2_O_3_ density of states (DOS), showing the band gap and the distribution of electronic states. (c) Electronic density of states (DOS) of CF5, demonstrating the effect of Co doping on electronic characteristics and band gaps.

Pristine α-Fe_2_O_3_ is an indirect bandgap semiconductor, according to the band structure calculations, with the conduction band minimum (CBM) at a separate *k*-point and the valence band maximum (VBM) at the *Γ*-point. The calculated bandgap, which is shown in Fig. 7(a), is 2.3 eV, which is the same value as previously published. The Fe 3d orbitals predominate in the conduction band, whilst the O 2p orbitals contribute to the majority of the valence band. The electrical structure is significantly influenced by the Fe 3d-O 2p hybridization.

The band structure changes noticeably when 5% Co is doped at Fe sites. The bandgap drops to 1.93 eV, suggesting the formation of impurity states close to the conduction band, as shown in Fig. 7(c). The Co 3d orbitals, which add more electronic channels and may improve electrical conductivity, are the source of these states. Moreover, a shift in the Fe 3d bands results from a minor perturbation of the Fe electronic states caused by Co inclusion.

Moreover, compared to normal LDA/GGA, which frequently underestimates the band gap, the LDA + U technique tends to increase it.^72,73^ Compared to the conventional DFT approaches, the use of U helps to correct the electron–electron interactions, especially in the Fe 3d orbitals, which results in a bigger bandgap and a more accurate representation of the electronic structure.^74^

### 3.4 DOS analysis

As shown in Fig. 7(b and c), the atom projected density of states (PDOS) offers vital information on the electrical structure of both pure and CF5. The O 2p states contribute to the majority of the valence band in pure α-Fe_2_O_3_, with Fe 3d states contributing close to the VBM. Fe 3d orbitals contribute to the conduction band, which has a wide band gap and restricts electrical conductivity. The DOS undergoes notable changes at 5% Co doping, especially in the vicinity of the CBM. By adding Co 3d states, more electronic states are produced close to the Fermi level, which lowers the bandgap and increases the concentration of carriers. This change suggests increased Fe–Co hybridization-induced electrical conductivity. Additionally, the doped system is better suited for applications needing effective charge transfer because the presence of Co states close to the CBM promotes electron transport.

**Fig. 8:**
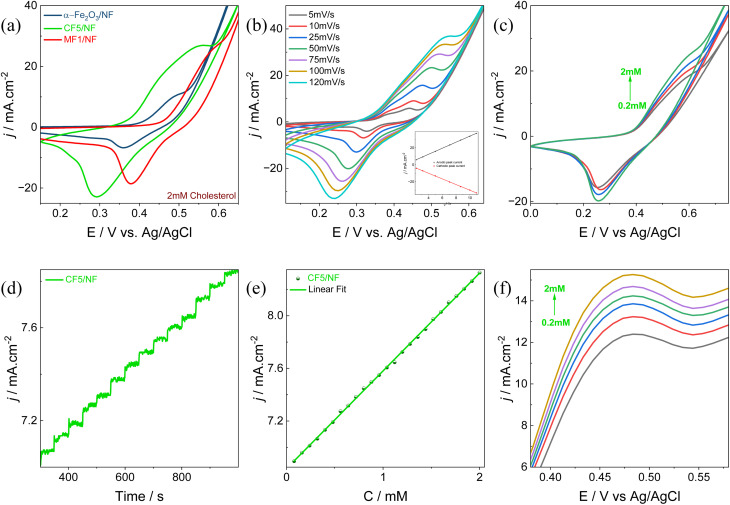
(a) Comparison of CVs for the influence of 2 mM cholesterol in 0.5 M KOH at a scan rate of 50 mV s^−1^ for α-Fe_2_O_3_/NF, CF5/NF, and MF1/NF; (b) scan rate study of CF5/NF in 0.5 M KOH from 5 to 120 mV s^−1^. The anodic and cathodic peak currents are inserted as a function of the square root of the scan rate (inset); (c) successive addition analysis for CF5/NF at a scan rate of 50 mV s^−1^; (d) CA study of CF5/NF conducted in a 0.5 M KOH solution, and the measurements performed under stirring conditions at an applied potential of +0.55 V; (e) calibration plot of CF5/NF to estimate the cholesterol levels; and (f) DPV study of CF5/NF to analyze the electrochemical response to successive cholesterol additions.

The CV analysis showed a progressive enhancement in both oxidation and reduction peak currents upon exposure to 2 mM cholesterol for α-Fe_2_O_3_/NF, CF5/NF, and MF1/NF in 0.5 M KOH electrolyte, at a scan rate of 50 mV s^−1^, as depicted in Fig. 8(a). In particular, the CF5/NF CV analysis with and without the addition of cholesterol at a scan rate of 50 mV s^−1^ was determined, as shown in Fig. S8(a), which reveals the efficiency of CF5/NF towards cholesterol sensing. The improved cholesterol-sensing performance of CF5/NF is ascribed to its well-defined crystalline structure, enhanced surface area, and superior electron transfer capabilities. Additionally, modifications in band gap energy, as indicated by the DFT study, contribute to a significant enhancement in electrocatalytic activity toward cholesterol oxidation. These factors collectively underscore the potential of CF5/NF for advanced cholesterol biosensing applications. Eqn (5)–(7) represent the detailed mechanism:^75^

5 Fe_2_O_3_ (III) + OH^−^ → Fe_2_O_3_ (III) − O_ads_

6 Fe_2_O_3_ (III) − O_ads_ → Fe_2_O_3_ (IV) = O + e^−^ + H^+^

7 Fe_2_O_3_ (IV) = O + cholesterol → cholest-4-en-3-one + Fe_2_O_3_ (III) − O_ads_ + e^−^ + H^+^

α-Fe_2_O_3_ contributes structural stability and a porous surface with an extensive surface area, making it an ideal host matrix. The mechanism can be illustrated as follows: Initially, as presented in eqn (5), the hydroxide ion (OH^−^) present in the electrolyte adsorbs onto the surface of Fe_2_O_3_, leading to the generation of adsorbed oxygen species (O_ads_) on Fe_2_O_3_ (iii). These adsorbed oxygen species undergo oxidation, leading to the formation of Fe_2_O_3_ (iv) with a higher oxidation state, along with the release of a proton (H^+^) and an electron (e^−^). Upon the addition of cholesterol to the electrolyte, Fe_2_O_3_ (IV) reacts and facilitates the formation of cholest-4-en-3-one. During this interval, Fe_2_O_3_ (iv) is reduced back to Fe_2_O_3_ (iii) with an adsorbed oxygen species, releasing additional e^−^ and H^+^. It was noted that the release of two electrons during this process aligns with the results obtained from the Laviron equation (eqn (8)):8
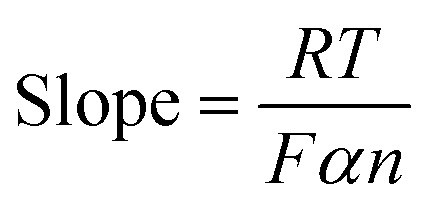


Fig. S7(a) gives the implementation of the Laviron equation, obtained by graphing the slope of ln (*ν*) against the oxidation peak potential (*E*). This equation involves the ideal gas constant (*R* = 8.314 J), faraday's constant (*F* = 96 480 C mol), temperature (*T* = 300 K), transfer coefficient (*α* = 0.5), and the number of electrons associated with cholesterol oxidation (*n*).^34^ The study showed that 1.72 electrons are exchanged during cholesterol oxidation, closely approximating a two-electron transfer mechanism, which is further corroborated by DFT analysis.

Fig. 8(b) illustrates the scan rate study of CF5/NF, encompassing a potential range from 5 mV s^−1^ to 120 mV s^−1^, and Fig. S8(b) depicts the scan rate study of α-Fe_2_O_3_/NF. With the increase in scan rate, there is a corresponding increase in the peak potentials of both oxidation and reduction processes. Under reduced scan rates, the formation of a diffusion layer between the electroactive species and the electrode surface results in reduced interaction, leading to lower anodic and cathodic peak currents. Conversely, an increase in scan rate leads to the breakdown of the diffusion layer, and at a certain scan rate, it vanishes. This results in a shift of the anodic peak towards more positive potentials and the cathodic peak towards more negative potentials with the increase in scan rates. Additionally, a linear correlation was observed between the square root of the scan rate and the redox peak currents of CF5/NF, as shown in the inset of Fig. 8(b), indicating that the formation of the diffusion layer impedes the interaction between electroactive species and the electrode surface, thereby facilitating electron transfer through an outer-sphere mechanism.

The subsequent study, illustrated in Fig. 8(c) and S8(c), presents the effects of successive additions of cholesterol within a linear concentration range of 0.2 mM to 2 mM in 0.5 M KOH, at a scan rate of 50 mV s^−1^ for CF5/NF and α-Fe_2_O_3_/NF, respectively. The data indicate a progressive rise in redox peak currents upon each incremental addition of cholesterol, signifying effective electrocatalytic oxidation and reduction of cholesterol. This observed enhancement in redox peak currents can be ascribed to the formation of a diffusion layer, facilitating improved interaction among the electroactive species and the electrode surface.

Fig. 8(d) depicts the CA current response of the CF5/NF electrode at an applied potential of +0.55 V in response to the sequential addition of cholesterol. Each addition of cholesterol results in a stepwise increase in current every 50 s, indicating the electrode's capacity to detect cholesterol effectively at each increment. Comparative CA responses for the α-Fe_2_O_3_/NF electrode are presented in Fig. S8(d). At the specified anodic peak potential, the absence of a diffusion layer allows direct interaction between the electroactive species and the electrode surface, resulting in a stepwise increase in current upon each cholesterol addition. Fig. 8(e) presents the calibration curve, demonstrating the linear response across a cholesterol concentration range from 0.2 mM to 2 mM. The CF5/NF electrode exhibited high sensitivity, measured at 1364.2 μA mM^−1^ cm^−2^ (±0.03, *n* = 3), as depicted in Table S3. Furthermore, the LOQ and LOD for the CF5/NF electrode were calculated to be ∼0.58 mM and ∼0.17 mM, respectively, with a response time of 2 s (Fig. S9(a)). In contrast, the α-Fe_2_O_3_/NF electrode demonstrated a sensitivity of 590 μA mM^−1^ cm^−2^ (±0.05, *n* = 3), as represented in Fig. S8(e) and Table S3.

Furthermore, DPV analysis was conducted to examine the electrochemical behavior of the CF5/NF electrode in the potential range of 0.35 to 0.65 V at a scan rate of 10 mV s^−1^ in 0.5 M KOH, as shown in Fig. 8(f). A notable increase in peak currents corresponded to the redox reactions as the cholesterol concentration increased, confirming the electrode's catalytic activity. These findings underscore that CF5/NF effectively responds to cholesterol due to its crystal structure, high surface area, excellent electron transfer, decrease in charge transfer impedance, increased surface active sites, and the defects of the CF5/NF electrode for cholesterol biosensing applications, demonstrating its potential for precise and dependable measurement of cholesterol levels.

**Fig. 9:**
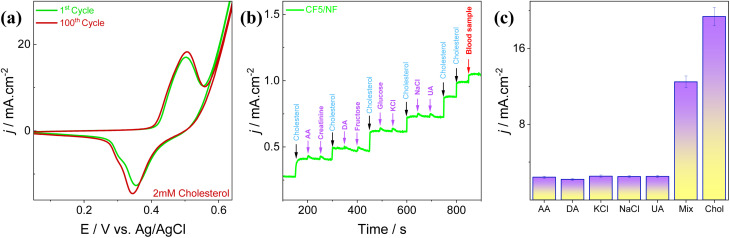
(a) CVs of CF5/NF for 100 cycles in a 0.5 M KOH solution containing 2 mM cholesterol, with an applied potential scan rate of 50 mV s^−1^; (b) interference study of CF5/NF in a 0.5 M KOH solution under stirring conditions to assess its reaction with various interfering species of 1 mM at a potential of +0.55 V; and (c) DPV interference study of CF5/NF at a scan rate of 10 mV s^−1^ and a pulse amplitude of 50 mV.

To confirm the long-term reproducibility of the CF5/NF electrode, CV for 100 cycles in 0.5 M KOH containing 2 mM cholesterol was performed. The resulting data, depicted in Fig. 9(a), exhibited minimal changes in oxidation and reduction peak potentials, retaining approximately 95% of its initial characteristics even after 100 cycles, while the α-Fe_2_O_3_/NF electrode retained 84.4% as presented in Fig. S8(f). Furthermore, the reproducibility of the CF5/NF electrode was assessed by testing three different electrodes in a 0.5 M KOH solution, as shown in Fig. S10(a), in which electrodes demonstrated consistent current ranges, thereby confirming their reproducibility. Additionally, the stability of the CF5/NF electrode was evaluated for 90 days, as depicted in Fig. S10(b), indicating that the electrode maintained stability with only a slight decrease in the current range, without any drastic changes. This indicates remarkable stability and reproducibility in the electrode's electrochemical performance. This study demonstrates that the CF5/NF electrode can maintain its catalytic activity and structural integrity through repeated use, underscoring its effectiveness for consistent and reliable cholesterol sensing.

One of the predominant characteristics of an effective biosensor is selectivity.^76,77^ To evaluate the selectivity of the CF5/NF electrode as a biosensor, an interference analysis was conducted using CA under stirring conditions at a constant potential of +0.55 V in 0.5 M KOH. The electrode was examined with various interfering species, including ascorbic acid (AA), creatinine, dopamine (DA), fructose, glucose, KCl, NaCl, and uric acid (UA), with two different concentrations signifying 0.5 mM and 1 mM of interfering species, as demonstrated in Fig. S9(a) and 9(b), respectively. The results indicated a stepwise increase in current exclusively upon adding cholesterol, with no significant current rise observed during the addition of the interfering species. This demonstrates that the CF5/NF electrode is highly selective for cholesterol biosensing. Moreover, an increase in current was observed upon adding a blood sample containing cholesterol, further confirming the electrode's sensitivity to cholesterol. To complement the interference study with CA, DPV analysis was also performed, as shown in Fig. 9(c). This analysis included the individual interfering species, a mixture of interfering species with cholesterol, and cholesterol alone. The results showed no significant interference from the interfering species in the mixture, while there was a noticeable increase in the current for cholesterol. These findings confirm the CF5/NF electrode's excellent sensing properties for cholesterol, demonstrating high selectivity, sensitivity, and reliability for cholesterol biosensing applications in comparison with those of existing metal oxide-based electrodes (Table 1).

**Table 1:** Comparison of the electrode biosensing capabilities of CF5/NF with those of the existing metal oxide-based cholesterol biosensors reported in the literature

Electrode material	Sensitivity (μA mM^−1^ cm^−2^)	LOD (mM)	Linear range (mM)	Ref
ZnO nanorods	4.2	1.78	1–9	78
Cu_2_O–MoS_2_	73.55	0.0036	0.0001–0.18	42
NiO/MoS_2_	7.95	0.209	0.259–3.88	79
Fe_3_O_4_@SiO_2_/MWNT	—	0.005	0.01–4	80
Oxidized Zn–In nanostructure	81	—	0.5–09	81
Calcein–Co_3_O_4_ NCs	—	0.00048	0.001–0.05	82
PMO-BMCP	226.30	0.00111	0.00002–333.3	83
CF5/NF	1364.2 (±0.03, *n* = 3)	∼0.17	0.2–2	This work

To further validate the performance of the CF5/NF electrode, we conducted real-sample analysis using known cholesterol concentrations in human blood serum collected from nearby hospitals. Initially, the electrode was tested with synthetically available cholesterol twice at a concentration of 2 mM and then with a real sample, as depicted in Fig. S9(b). For the real-sample analysis, the data obtained were compared with theoretical values provided by the hospitals. As summarized in Table 2, the results showed an accuracy level of approximately 94–98%. This high degree of accuracy underscores the CF5/NF electrode's excellent sensing properties for cholesterol, confirming its potential for practical application in clinical diagnostics and real-world cholesterol analysis.

**Table 2:** Analysis of human blood serum for cholesterol testing

Patient	Gender	Clinically tested result (mg dL^−1^)	Experimental result (mg dL^−1^)	% accuracy
Patient 1	Female	308	291.6	94.68%
Patient 2	Female	182	174.7	96%
Patient 3	Male	211	201.2	95.36%
Patient 4	Male	202	195.3	96.66%
Patient 5	Female	200	196	98%
Patient 6	Female	260	252.2	97%

**Fig. 10:**
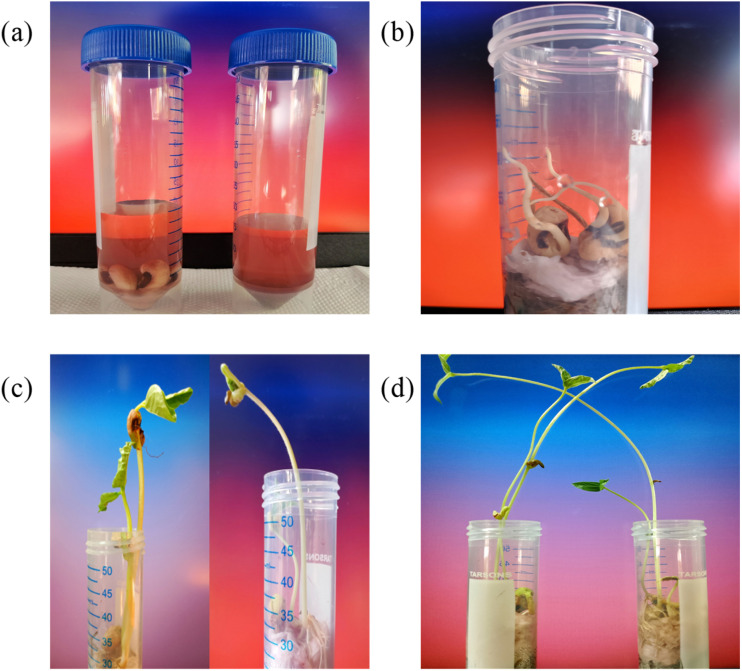
Preliminary study of biocompatibility demonstrating enhanced plant growth using CF5 solution, with photographs demonstrating seed germination on (a) day 1; (b) day 3; (c) day 8; and (d) day 13.

In this work, a primary investigation towards the non-toxic behavior and biocompatibility of CF5 was performed *via* plant growth in addition to an electrochemical investigation. The CF5 solution is simply made by dissolving the synthesized product in water, highlighting its low environmental impact, as shown in Fig. 10(a–d). Green gram and horse gram seeds were selected as the subjects for the study. The seeds were initially immersed in the solution, and by day 3, germination was observed, as shown by the appearance of tiny buds. By day 13, healthy seedling growth was noticeable.^84^ These outcomes underscore that the material CF5 does not exhibit any phytotoxicity and demonstrate the importance of examining the non-toxic behavior of nanomaterials, particularly when aimed at biosensing applications.

## 4 Conclusions

This study successfully synthesized α-Fe_2_O_3_ and M_*x*_Fe_2−*x*_O_3_ (M = Mn–Co and *x* = 0.01, 0.05, and 0.1) and fabricated a working electrode by coating the materials on an NF substrate. Comprehensive structural, optical, morphological, and electrochemical analyses identified CF5/NF as a superior electrode material, exhibiting excellent sensitivity, a high surface area, and efficient electron transfer. The CF5/NF electrode demonstrated remarkable performance in cholesterol sensing, with a linear detection range from 0.2 mM to 2 mM, a sensitivity of 1364.2 μA mM^−1^ cm^−2^ (±0.03, *n* = 3) in 0.5 M KOH, an LOD of ∼0.17 mM, and an LOQ of ∼0.58 mM. Additionally, the electrode exhibited a rapid response time of 2 s. The evaluation of real samples revealed that the CF5/NF electrode exhibited outstanding cholesterol-sensing capabilities, demonstrating a negligible interference from other species and underscoring its potential for enzyme-free cholesterol detection. The study concludes that the CF5 nanocomposite is a prominent nanocomposite for cholesterol sensing due to its robust electrochemical characteristics, non-toxic behavior, biocompatibility, and efficacy in detecting cholesterol in human blood samples, signifying a viable tool for clinical and pharmaceutical applications. This research shows good results over laboratory-scale synthesis; expanding on this information, the future research is mainly focused on scaling up the synthetic procedure for a miniaturized and wearable platform for real-time point-of-care cholesterol sensing.

## Ethical approval

The utilization of human serum samples in this research was authorized by the Ethical Committee of the National Institute of Technology Karnataka, Surathkal, under reference number NITK/Bioethics/2023/02, dated 24 April 2023. Experimental protocols were meticulously followed in accordance with institutional regulations and legal frameworks, ensuring ethical research conduct.

## Author contributions

Sushmitha S: writing – original draft, visualization, methodology, investigation, conceptualization. Subhasmita Ray: writing – review & editing, visualization, software. Lavanya Rao: writing – review & editing, visualization, methodology, investigation, conceptualization. Mahesha P Nayak: visualization, investigation. Karel Carva: writing – review & editing, supervision, software. Badekai Ramachandra Bhat: writing – review & editing, supervision, project administration.

## Conflicts of interest

The authors declare that they have no known competing financial interests or personal relationships that could have appeared to influence the work reported in this paper.

## Supplementary Material

RA-015-D5RA04373E-s001

## Data Availability

Additional relevant data supporting the findings of this study are available from the corresponding author upon reasonable request. All characterization data and raw experimental results are included within the manuscript and SI. See DOI: https://doi.org/10.1039/d5ra04373e.
