# Supplementary material for: Electrochemical insights into manganese–cobalt doped α-Fe2O3 nanomaterial for cholesterol detection: a comparative approach
Source: RSC Adv. 2025 Sep 18;15(41):34176–90. doi: 10.1039/d5ra04373e (PMC12444626; doi:10.1039/d5ra04373e)
Supplement: RA-015-D5RA04373E-s001 [file RA-015-D5RA04373E-s001.pdf]

Supplementary Information (SI) for RSC Advances.
This journal is © The Royal Society of Chemistry 2025

ELECTROCHEMICAL INSIGHTS INTO MANGANESE-COBALT DOPED α-Fe_2O_3 NANOMATERIAL FOR CHOLESTEROL DETECTION: A COMPARATIVE APPROACH

Sushmitha S [a], Subhasmita Ray [b], Lavanya Rao [a], Mahesha P Nayak [a], Karel Carva [b], Badekai Ramachandra Bhat [a]*

[a] Department of Chemistry, Catalysis and Materials Chemistry Laboratory, National Institute of Technology Karnataka, Surathkal, D.K., Karnataka 575 025, India.*

[b] Department of Condensed Matter Physics, Faculty of Mathematics and Physics, Charles University, Ke Karlovu 3, Prague 12116, Czech Republic.

**Corresponding Author: ram@nitk.edu.in*

1. Results and discussions:

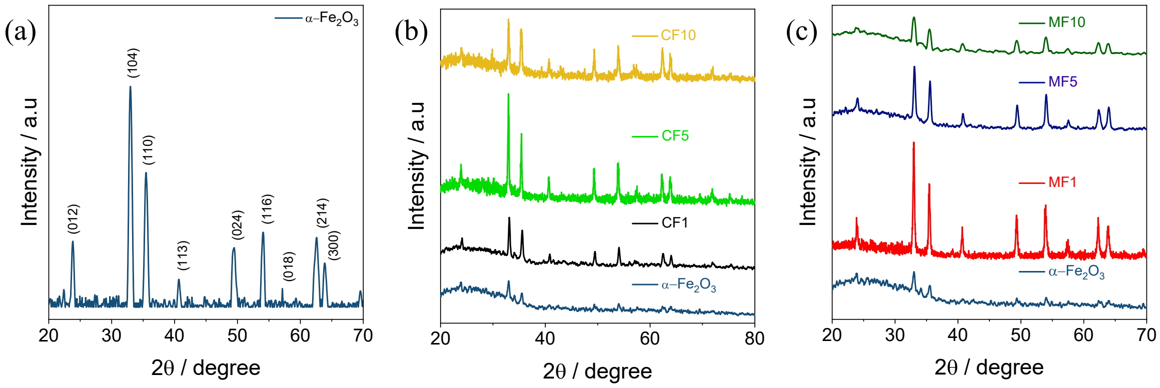

Figure. S1: *XRD analysis of (a) α-Fe_2O_3; (b) α-Fe_2O_3 doped with cobalt; (c) α-Fe_2O_3 doped with manganese, respectively.*

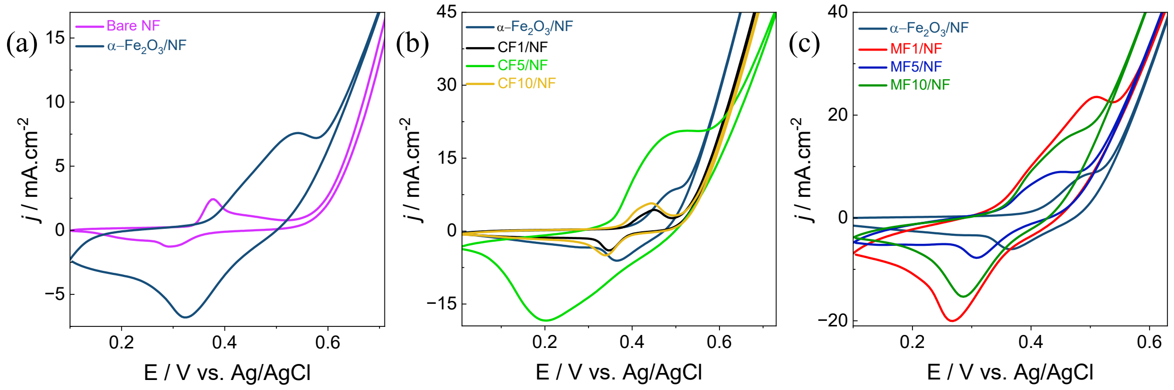

Figure. S2: *Comparison of CVs of (a) bare NF and α-*Fe_2O_3*/NF; (b) α-*Fe_2O_3*/NF with cobalt-doped nanocomposite; (c) α-*Fe_2O_3*/NF with manganese-doped nanocomposite.*

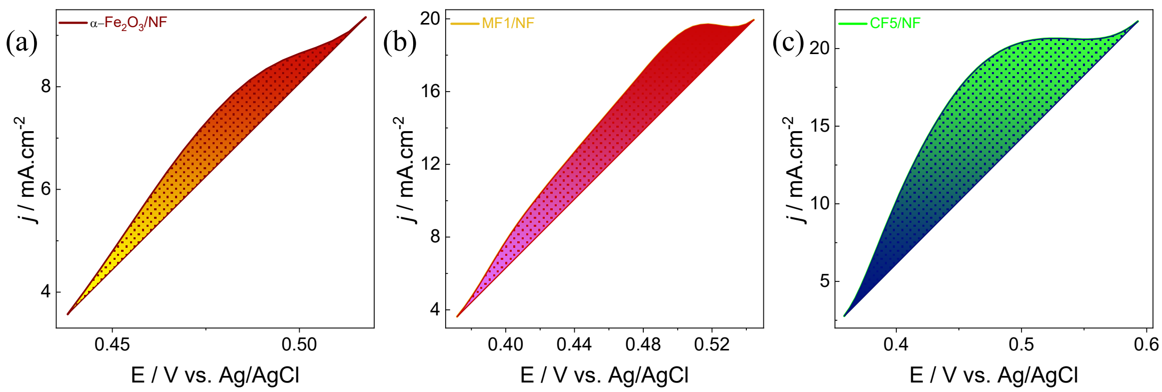

Figure. S3: *Oxidation peak study of (a) α-Fe_2O_3/NF; (b) MF1/NF; (c) CF5/NF.*

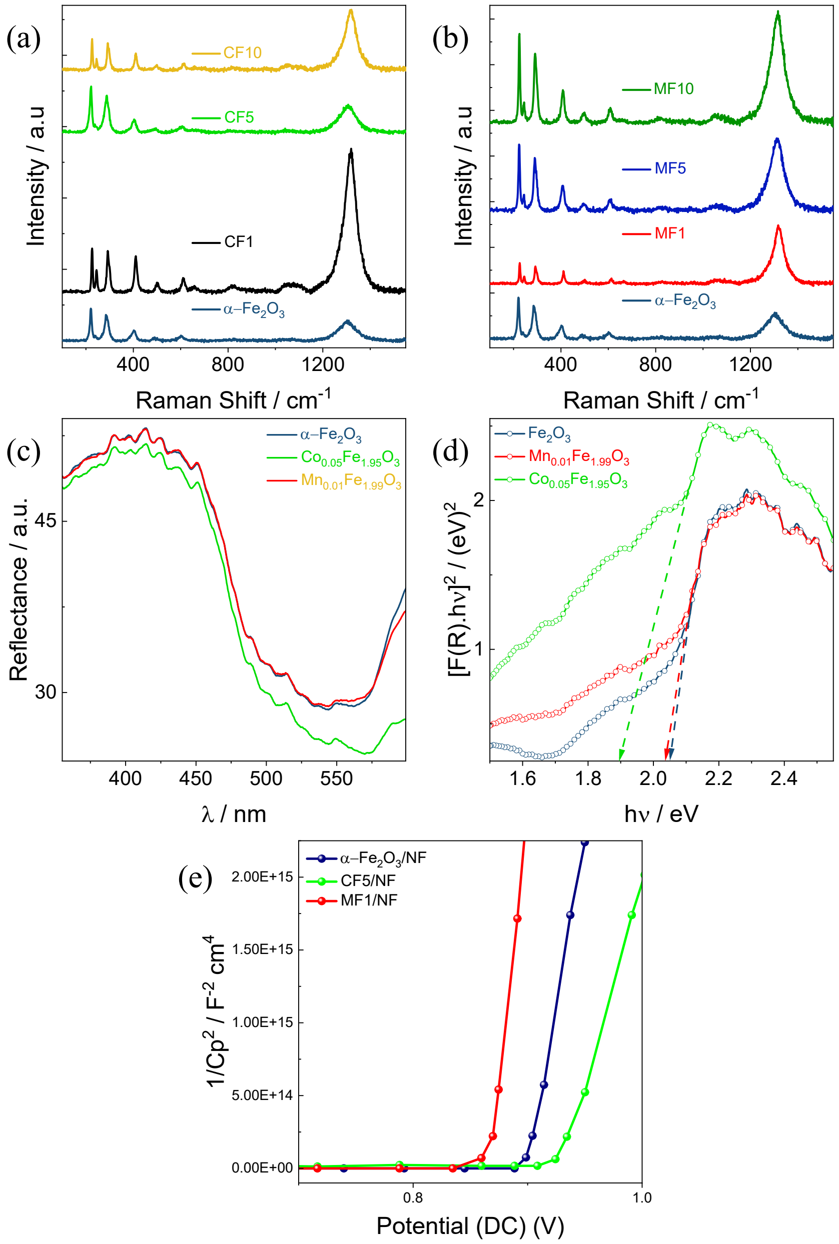

Figure. S4: *Raman spectroscopy analysis of (a) Cobalt-doped α-Fe_2O_3; (b) Manganese-doped α-Fe_2O_3; (c) Reflectance spectra of α-Fe_2O_3, CF5, and MF1, respectively; (d) Optical band gap plots obtained from UV spectra for α-Fe_2O_3, CF5, and MF1; (e) Mott Schottky plot of α-Fe_2O_3/NF, CF5/NF, and MF1/NF respectively.*

The Mott–Schottky (MS) analysis provides further clarification of the apparent discrepancy between the optical band gap and EIS results. In MF1, despite the marginal reduction in band gap, the steeper MS slope signifies a lower donor density and reduced concentration of mobile carriers. These defect-related states primarily function as trap centres, thereby accounting for the increased charge-transfer resistance. In contrast, CF5 exhibits a comparatively flatter MS

slope, indicative of a higher donor density arising from Co-induced oxygen vacancies, which enhance electronic conductivity. Collectively, the MS results underscore that interfacial charge-transfer kinetics are more strongly influenced by carrier density and defect chemistry than by band gap modulation alone.

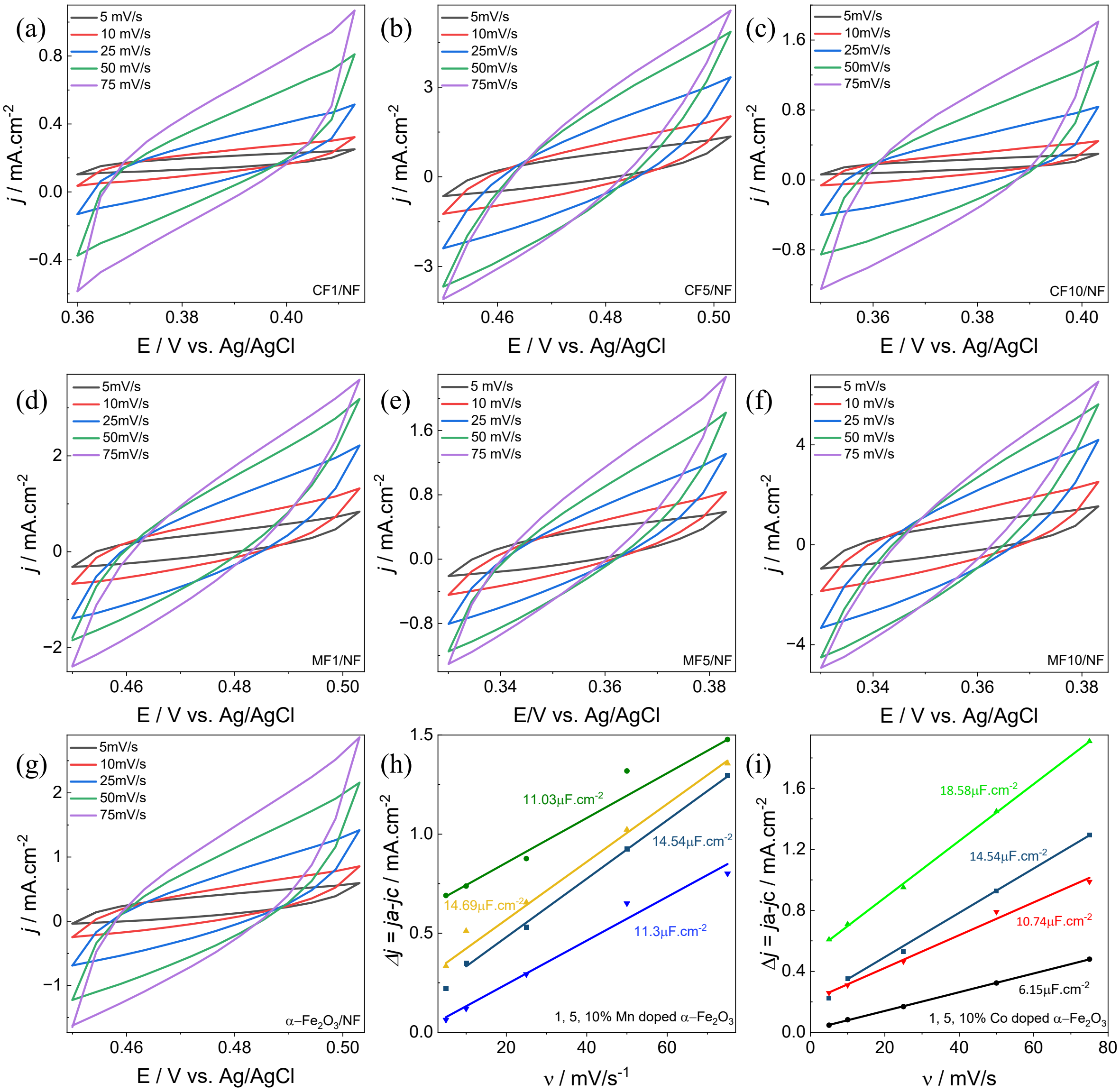

Figure. S5: *ECSA analysis of (a) CF1/NF; (b) CF5/NF; (c) CF10/NF, (d) MF1/NF (e) MF5/NF (f) MF10/NF (g) α-Fe_2O_3/NF (h, i) Calibration plot of doped Mn and Co with α-Fe_2O_3, respectively.*

Material	Slope (μF.cm^{-2})	(Slope / 40 × 2) (cm^2)
**α-Fe_2O_3/NF**	**14.54**	**0.182**
**MF1/NF**	**14.69**	**0.184**
MF5/NF	11.3	0.141
MF10/NF	11.03	0.138
CF1/NF	6.15	0.077
**CF5/NF**	**18.58**	**0.232**
CF10/NF	10.74	0.134

Table. S1: *Surface Area Calculation*

**Parameter**	**α - Fe_2O_3/NF**	**CF5/NF**	**MF1/NF**
R_s (Ω)	0.7395	0.7171	1.148
R_{ct} (Ω)	1.791	1.637	3.995
Q_{ct} (μF)	11.11	11.62	4.101
R_{ce} (Ω)	80.4	126.1	120.6
Q_{ce} (mF)	0.455	0.251	0.381

Table. S2: *EIS parameters for the fitted Circuit*

(a)

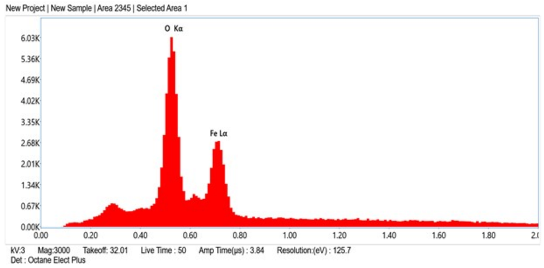

Element	Weight %	Atomic %
O K	57.8	82.7
Fe L	42.2	17.3

(b)

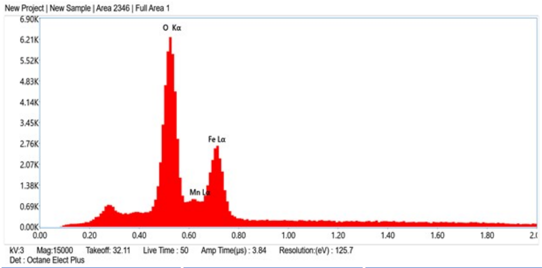

Element	Weight %	Atomic %
O K	60.6	84.3
Mn L	0.2	0.1
Fe L	39.3	15.6

(c)

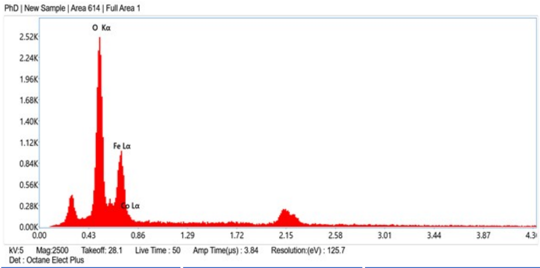

Element	Weight %	Atomic %
O K	55.7	81.5
Fe L	43.9	18.4
Co L	0.5	0.2

Figure. S6: *EDAX analysis of (a) α-Fe_2O_3; (b) MF1; (c) CF5.*

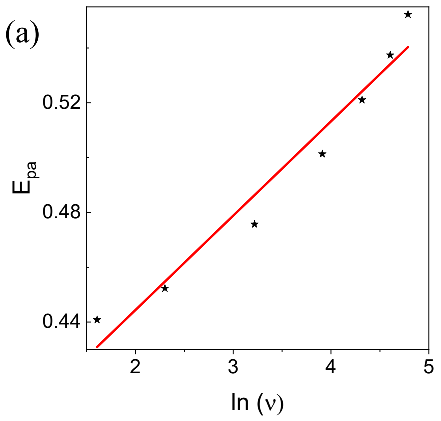

Figure. S7: *(a) Laviron Equation for CF5/NF.*

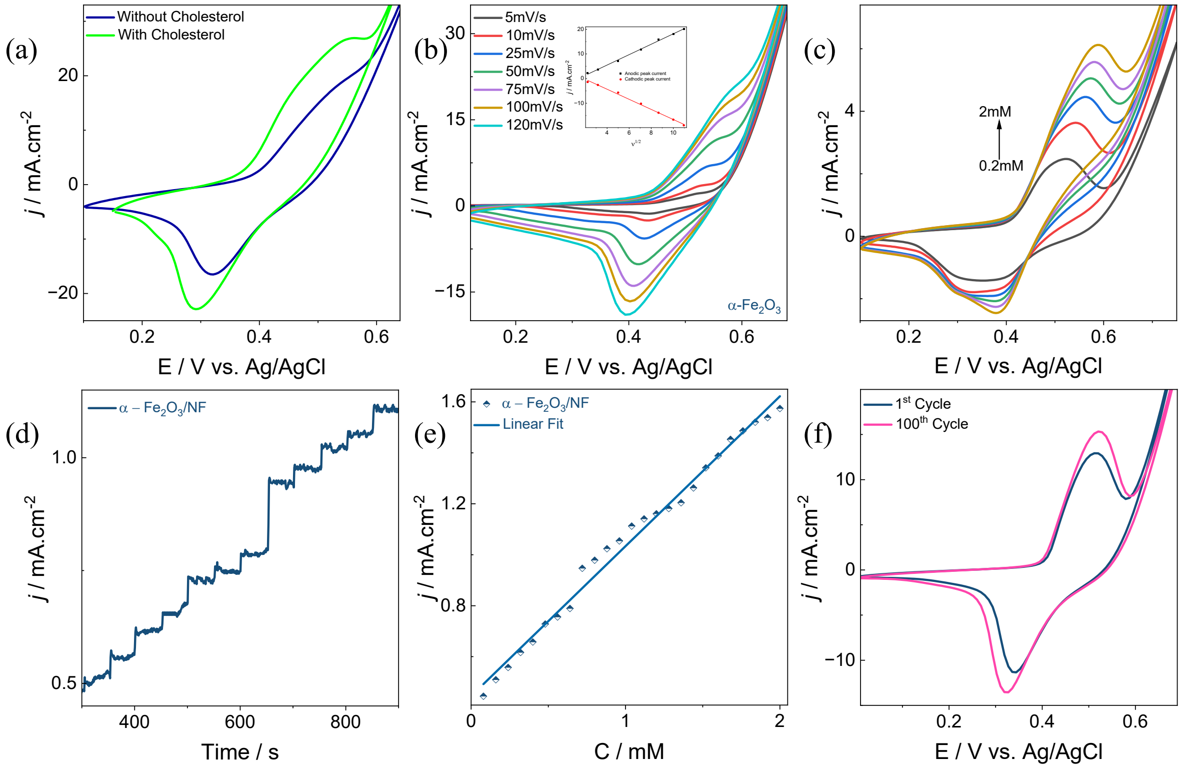

Figure. S8: *(a) CV comparison of CF5/NF with and without Cholesterol. (b) Scan rate study of α-Fe_2O_3/NF; (c) Successive Addition of 2 mM Cholesterol in 0.5 M KOH; (d) CA study of α-Fe_2O_3/NF; (e) Calibration cure of α-Fe_2O_3/NF; (f) CVs of α-Fe_2O_3/NF for 100 cycles in 0.5 M KOH with 2 mM Cholesterol.*

(a)

No. of trials	Material	Sensitivity (μA.mM^{-1}.cm^{-2})
1	CF5/NF	1364.17
2	CF5/NF	1364.23
3	CF5/NF	1364.20
**Mean ± SD**		**1364.20 ± 0.03**

(b)

No. of trials	Material	Sensitivity (μA.mM^{-1}.cm^{-2})
1	α-Fe_2O_3/NF	589.95
2	α-Fe_2O_3/NF	590.05
3	α-Fe_2O_3/NF	590.00
**Mean ± SD**		**590.00 ± 0.05**

Table. S3: *Reproducibility of (a) CF5/NF and (b)* α-Fe_2O_3/NF *sensitivities over three trials, expressed as mean ± SD*

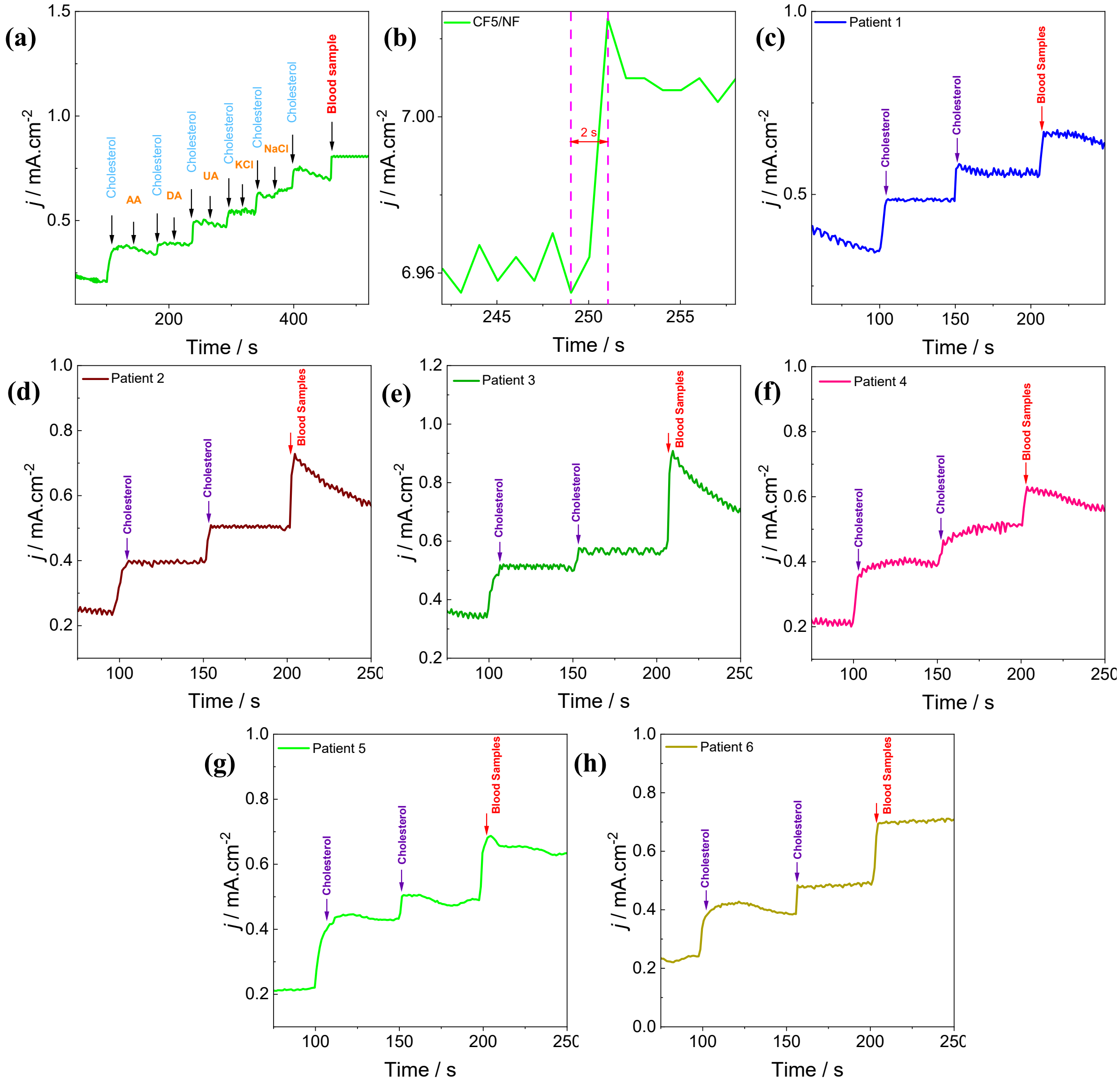

Figure. S9: *(a) An interference study of CF5/NF in a 0.5 M KOH solution under stirring conditions to assess its reaction to various interfering species of 0.5mM at a potential of +0.55 V;(b) Response time plot using CA; (c-h) CA analysis of real human blood serum (Red) and synthetic cholesterol (Lavender) of 6 patients.*

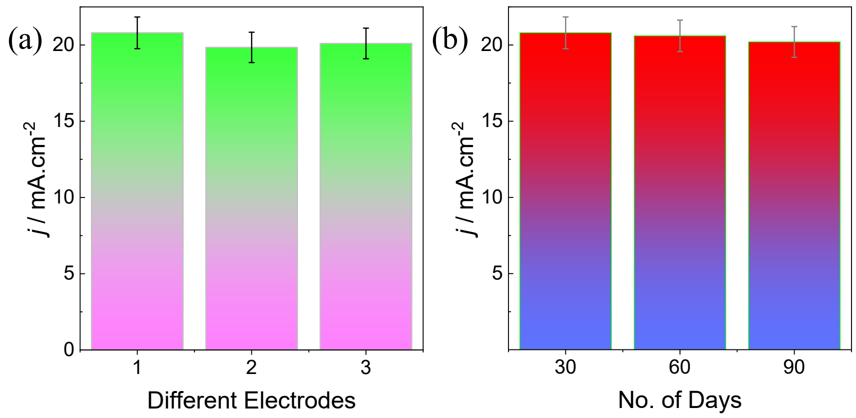

Figure. S10: *(a) Reproducibility was assessed using three different CF5/NF electrodes; (b) The stability of the CF5/NF electrode was evaluated for 90 days.*
